# Functional assessment of antibody oxidation by native mass spectrometry

**DOI:** 10.1080/19420862.2015.1052199

**Published:** 2015-05-22

**Authors:** Markus Haberger, Anna-Katharina Heidenreich, Tilman Schlothauer, Michaela Hook, Jana Gassner, Katrin Bomans, Michelle Yegres, Adrian Zwick, Boris Zimmermann, Harald Wegele, Lea Bonnington, Dietmar Reusch, Patrick Bulau

**Affiliations:** 1 Pharma Technical Development Penzberg; Roche Diagnostics GmbH; Penzberg, Germany; 2 Biochemical and Analytical Research; Large Molecule Research; Roche Pharma Research and Early Development; Roche Innovation Center; Penzberg, Germany; 3 Pharma Biotech Penzberg; Roche Diagnostics GmbH; Penzberg, Germany

**Keywords:** native mass spectrometry, protein degradation, oxidation, recombinant antibodies, neonatal Fc receptor, critical quality attributes

## Abstract

Oxidation of methionine (Met) residues is one of several chemical degradation pathways for recombinant IgG1 antibodies. Studies using several methodologies have indicated that Met oxidation in the constant IgG1 domains affects in vitro interaction with human neonatal Fc (huFcRn) receptor, which is important for antibody half-life. Here, a completely new approach to investigating the effect of oxidative stress conditions has been applied. Quantitative ultra-performance liquid chromatography mass spectrometry (MS) peptide mapping, classical surface plasmon resonance and the recently developed FcRn column chromatography were combined with the new fast-growing approach of native MS as a near native state protein complex analysis in solution. Optimized mass spectrometric voltage and pressure conditions were applied to stabilize antibody/huFcRn receptor complexes in the gas phase for subsequent native MS experiments with oxidized IgG1 material. This approach demonstrated a linear correlation between quantitative native MS and IgG-FcRn functional analysis.

In our study, oxidation of the heavy chain Met-265 resulted in a stepwise reduction of mAb3/huFcRn receptor complex formation. Remarkably, a quantitative effect of the heavy chain Met-265 oxidation on relative binding capacity was only detected for doubly oxidized IgG1, whereas IgG1 with only one oxidized heavy chain Met-265 was not found to significantly affect IgG1 binding to huFcRn. Thus, mono-oxidized IgG1 heavy chain Met-265 most likely does not represent a critical quality attribute for pharmacokinetics.

## Abbreviations

MS: mass spectrometry
huFcRn: human neonatal Fc receptor
Met: methionine
SPR: surface plasmon resonance
mAbs: monoclonal antibodies
CDR: complementary-determining region
LC-MS: liquid chromatography-mass spectrometry

## Introduction

Oxidative degradations that occur in bio-therapeutics have been extensively reviewed.^1-7^ Recombinant monoclonal antibodies (mAbs) are exposed to process and storage conditions that might influence the rate and extent of these modifications.^8^

Oxidation of methionine (Met) can be induced by incubation with oxidizing agents like H_2_O_2_^5,6,9-13^ or tert-butylhydroperoxide (TBHP),^6,9,10,14-17^ by ultraviolet light irradiation,^17,18^ and it is also observed in pharmaceutical antibodies under elevated temperature conditions.^10,14,18,19^ Tryptophan (Trp) residues have been oxidized with oxygen radicals^20^ or with Fe(II)/EDTA/Asc,^21^ and the oxidation of Trp residues in mAbs has also been reported recently using ozone and UV irradiation.^17,22^ Significant Trp oxidation in parathyroid hormone (PTH) was also reported by Ji et al., who used 2,2′-azobis(2-amidinopropane) dihydrochloride (AAPH) as the oxidizing agent.^5^ So far, however, only one example of an oxidation-susceptible Met residue within the complementary-determining regions (located in the heavy chain CDR 3) of recombinant IgG1 antibodies has been reported.^19^ In contrast, the heavy chain Met-107 (CDR 3) of trastuzumab was found to be not susceptible to oxidation.^16^ Induction of Trp oxidation in the CDRs (heavy chain Trp-105; CDR 3) of a mAb by photo-oxidation resulted in a progressive loss of target binding and biological activity.^17^ In another case, the light chain Trp-32 (CDR 1) of a recombinant IgG1 was found to be susceptible to oxidation under elevated temperature conditions over time.^10^

Oxidation of Met residues in the constant domains of recombinant IgG1 antibodies has been demonstrated to affect the in vitro interaction with Protein A, the neonatal Fc receptor, and binding to the Fcγ receptors.^9,23,24^ Recently, a clear effect of Met oxidation in the constant region of an IgG1 on the pharmacokinetics has been reported in 2 independent in vivo studies.^13,25^

For large biomolecules such as recombinant antibodies, bottom-up liquid chromatography-mass spectrometry (LC-MS) of proteolytic peptides is often the method of choice for monitoring site-specific oxidation reactions.^2,5,9-13,17,19,22,26^ However, advances in native mass spectrometry has enabled the analysis of intact protein and protein complexes under more physiological conditions.^27,28^ During recent years, several authors have successfully demonstrated the application of native MS for the qualitative and quantitative structural characterization of recombinant antibodies and new therapeutic protein formats.^29-38^ Moreover, native MS also allows the analysis of dimer formation, antibody aggregation, and antibody-antigen binding.^39-41^

For our study, an approach employing oxidative stress conditions and quantitative LC-MS peptide mapping combined with native MS for the simultaneous induction, quantification and functional assessment of Met oxidation in recombinant antibodies was developed. This test system enabled us to study the effect of Met oxidation in the constant IgG domains on in vitro binding to the neonatal Fc receptor.

## Results

An approach employing native electrospray ionization (ESI)-MS conditions was used to study the effect of Met oxidation in the constant IgG domains on in vitro binding to the human neonatal Fc receptor (huFcRn). Several mAb-:huFcRn receptor ratios (2:1, 1:1, 1:2, and 1:3) were tested. Due to the relative weak binding^42-44^ of the antibody to the huFcRn receptor a ratio of 1:3 was identified as most suitable condition for all further native ESI-MS experiments (data not shown). The ESI-MS instrument parameters were first optimized for the detection of the intact antibody (mAb3) and subsequently for the mAb3/huFcRn receptor complexes.

**Figure 1.**
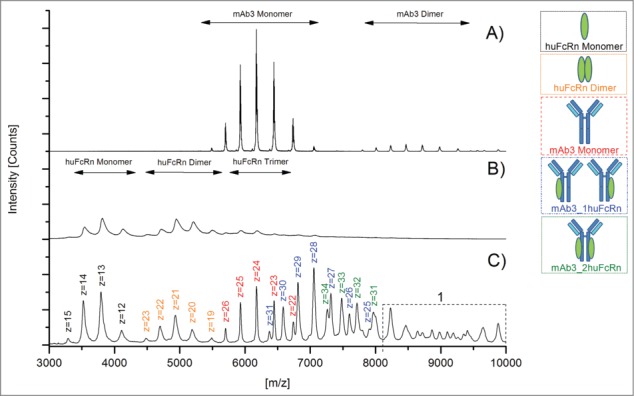
Native MS spectra of mAb3/huFcRn solutions recorded with optimized voltage and pressure parameter settings. (**A**) mAb3 alone, (**B**) huFcRn alone, and (**C**) mAb3/huFcRn solution (ratio 1:3). z, charge state; 1, higher order complexes of mAb3/huFcRn.

A mAb3/huFcRn solution (ratio 1:3) was analyzed with initial voltage parameters as recommended by the manufacturer (Cone 45V, RF Lens1 90V, Collision cell 30V). The initial pressure parameters (ESI-Source Pirani Guage backing pressure 2.7 mbar, Collision cell pressure 5.7 e^−3^ mbar) resulted in an analyzer penning value of 1.4 e^−4^ mbar and a time-of-flight (TOF) penning value of 7.3 e^−7^ mbar, respectively. Using these parameter settings only low sensitivity for protein signals above the mass to charge (*m/z*) ratio of 4500 was observed (data not shown). Consequently, the effect of all voltage and pressure parameters on the detection of higher *m/z* ions (corresponding to lower charge states) were tested as outlined in **Table 1**. Variation of the Cone and Collision cell voltages did not result in a higher sensitivity for signals above *m/z* 4500 (data not shown). However, the reduction of the pressure in the collision cell to 1.2 e^−2^ mbar did significantly stabilize the higher *m/z* ions in general, including the mAb3/huFcRn receptor complexes (data not shown). In addition, elevation of the RF Lens1 voltage from 90V up to 150 V also increased the sensitivity for protein signals above *m/z* 6500. **Figure 1** shows native MS spectra of mAb3 alone (**Fig 1A**), huFcRn alone (**Fig 1B**), and mAb3/huFcRn solution (ratio 1:3) recorded with optimized MS parameter settings (**Table 1**). Moreover, the optimized voltage and pressure parameters did not significantly affect the mass analyzer and TOF penning values and were applied for all subsequent studies with triple A mutant and oxidized mAb3 variants.

**Table 1.** Summary and evaluation of the MS voltage and pressure parameters tested for the native MS analysis of mAb3/huFcRn complexes

Parameter	Test range	Scope	Impact	Final Setting
Cone Voltage	35–100 V	Stabilization of complexes	No significant effect	45 V
RF Lens1 Voltage	0–200 V	Improved transport of the complexes in vacuum to enable better detection	A higher voltage resulted in an improved complex transport. Caused fragmentation at 200 V	150 V
Collision Cell Pressure	8e^−6^ bar – 2e^−5^ bar	Stabilization of complexes and limitation of dimer formation	Pressure reduction stabilized complex formation. Dimer formation at low pressure values	1e^−5^ bar
Pirani Guage Backing Pressure	2,0–2,7 bar	Limitation of dimer formation	Reduced dimer formation at higher pressure values	2,5 −2,7 bar
Collision Cell Voltage	0–100 V	Improved complex detection	No significant effect on complex stabilization but fragmentation at high voltage values.	20 V

**Figure 2.**
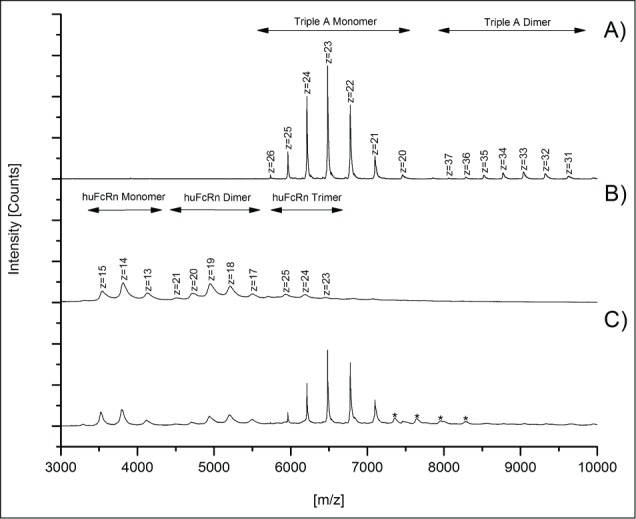
Native MS spectra of triple A IgG1/huFcRn solutions (ratio 1:3) to demonstrate the specificity of mAb3/huFcRn interaction. Spectra achieved with (**A**) triple A IgG1 mutant alone, (**B**) huFcRn alone, and (**C**) triple A IgG1/huFcRn solutions (ratio 1:3). Low abundant signals for non-specific A IgG1/huFcRn interactions are marked with asterisks (*).

The huFcRn receptor monomer was detected over the *m/z* range of 3000 to 4500, the selected antibody mAb3 monomer between *m/z* 5500 to 7000 and the mAb3/huFcRn receptor complexes (containing one or 2 FcRn molecules) were traceable between *m/z* 6000 and 8000 (**Figs. 1 and 2**). All selected charge states for mass determination and subsequent semi-quantitative functional analysis are summarized in **Table 2**. Under the applied native MS conditions, the semi-quantitative data suggests that mAb3/huFcRn receptor complexes with one FcRn represents the predominant form (50%), whereas non-complexed mAb3 (23%) and mAb3/huFcRn receptor complexes with 2 FcRn molecules (28%) were found at lower levels (**Table 3**). The observed ratio was not affected by varying cone voltage (as outlined in **Table 1**) suggesting that the relatively high abundance of mAb3/huFcRn receptor complexes with a 1:1 stoichiometry is due to an incomplete complex formation reaction rather than the result of a partial fragmentation of a weakly bound complex with 1:2 stoichiometry in the ion source region. However, by applying the described parameter settings, huFcRn and mAb3 dimer formation was observed (**Figs. 1 and 2**; **Table 2**), between *m/z* 4500 - 5500 and *m/z* 8000 and 9500, respectively. Moreover, the appearance of higher order aggregates with mass values beyond 250000 Da (*m/z* > 9000) was verifiable (**Fig 1**). The scientific evaluation of this observation is included in the discussion section.

**Table 2.** Outline of selected charge states for mass determination and subsequent semi-quantitative functional analysis of mAb3/huFcRn complexes. 1, Sum (∑) of charge state area intensities from the 3 most abundant charge states (in boldface) were selected for the semi-quantitative evaluation of non-complexed mAb3 and mAb3/huFcRn complexes with one or 2 FcRn molecules, respectively. 2, due to the mAb3 and huFcRn glycosylation heterogeneity the theoretical mass remained unknown. Therefore, the experimental mass was used as the theoretical mass

	charge state	*m/z*	area	∑ area^1)^	experimental mass [Da]	mean experimental. mass [Da]	theoretical mass [Da]
huFcRn	15	3284	482	13746	49242	49277	49277^2)^
	14	3520	5675		49265		
	13	3793	6575		49295		
	12	4110	1496		49305		
huFcRn Dimer	22	4481	360	7230	98556	98584	98554
	21	4697	1976		98619		
	20	4930	3548		98579		
	19	5189	1706		98580		
mAb3	26	5700	860	8644	148177	148196	148196^2)^
	25	5929	2586		148194		
	24	6176	3678		148189		
	23	6444	2380		148197		
	22	6738	1414		148222		
mAb3 + 1xhuFcRn	31	6373	911	17064	197526	197494	197473
	30	6584	3115		197486		
	29	6811	5687		197478		
	28	7054	7377		197476		
	27	7315	4000		197483		
	26	7598	2182		197516		
mAb3 + 2xhuFcRn	34	7260	3511	10915	246789	246814	246836
	33	7481	4112		246825		
	32	7714	3293		246827

**Table 3.** Identification and evaluation of mAb3 Met oxidation sites using oxidative stress conditions and quantitative UPLC-MS. Relative quantification (in %) was conducted by specific ion current chromatogram analysis of tryptic peptides using the GRAMS/32TM quantification software. Formation of mAb3 fragments and aggregates was monitored by size exclusion chromatography (SEC). FcRn binding activity was assessed by native ESI-MS, SPR-analysis, and analytical huFcRn affinity chromatography. All experiments were performed in triplicate (n = 3). SPR sensorgrams and huFcRn affinity chromatograms are depicted in the supplementary data. LC, light chain; HC, heavy chain; Asn, asparagine; Asu, succinimide; Asp, aspartate; deamid, total Asp/iso-Asp content; ox, oxidation; n.d., not detectable

	Oxidative stress condition				
Parameter	0% H_2_O_2_	0.003% H_2_O_2_	0.009% H_2_O_2_	0.015% H_2_O_2_	0.020% H_2_O_2_
LC-Asn-30					
LC-Asu-30	0.8 (<0.1)	1.0 (0.1)	1.3 (<0.1)	1.2 ((<0.1)	1.1 (<0.1)
LC-deamid-30	9.3 (0.1)	10.5 (0.2)	11.9 (0.2)	11.3 (0.3)	11.0 (0.1)
HC-Asn-54					
HC-Asu-54	5.0 (0.1)	5.4 (0.1)	5.6 (0.3)	5.6 (0.2)	5.5 (0.1)
HC-deamid-54	1.8 (0.1)	2.1 (<0.1)	2.2 (<0.1)	2.2 (<0.1)	2.2 (0.1)
HC-Asp-98					
HC-Asu-98	n.d.	n.d.	n.d.	n.d.	n.d.
HC-iso-Asp-98	3.9 (0.8)	4.3 (1.0)	4.7 (0.9)	5.7 (0.5)	4.6 (0.8)
HC-Asn-410,417,418					
HC-deamid-410,417,418	1.2 (0.1)	1.4 (0.1)	1.5 (0.2)	1.3 (0.1)	1.5 (0.2)
LC-Met-4					
LC-Met-ox-4	1.6 (0.2)	3.4 (0.4)	4.5 (0.4)	7.1 (0.4)	8.6 (0.9)
HC-Met-82					
HC-Met-ox-82	n.d.	n.d.	n.d.	n.d.	n.d.
HC-Met-100c					
HC-Met-ox-100c	5.7 (1.2)	5.1 (0.7)	12.3 (1.5)	17.4 (2.9)	21.8 (2.6)
HC-Met-265					
HC-Met-ox-265	3.5 (0.2)	22.5 (0.3)	46.3 (1.2)	60.9 (0.3)	71.2 (0.1)
HC-Met-459					
HC-Met-ox-459	2.2 (0.2)	7.4 (0.4)	17.4 (0.5)	28.1 (0.7)	34.6 (0.9)
SEC					
% Fragment	<0.1	<0.1	<0.1	<0.1	<0.1
% Monomer	99.9	99.9	99.9	99.9	99.9
% Aggregate	<0.1	<0.1	<0.1	<0.1	<0.1
Native MS					
% Free mAb3	23.0 (1.6)	26.5 (1.2)	30.4 (0.8)	36.6 (1.2)	44.5 (1.4)
% mAb_1xFcRn Complex	49.5 (2.6)	48.2 (0.7)	47.9 (0.9)	45.1 (1.8)	41.9 (3.1)
% mAb_2xFcRn Complex	27.6 (2.1)	25.3 (0.6)	21.5 (1.2)	18.3 (0.6)	13.7 (1.8)
SPR					
% FcRn binding	100.2 (0.2)	95.1 (1.2)	81.7 (1.8)	67.4 (2.1)	54.5 (0.2)
huFcRn affinity chromatography					
% Main Peak	100.0 (0.0)	71.1 (1.2)	29.1 (0.9)	10.4 (0.1	4.44 (0.5)
% Prepeak1		26.7 (1.0)	51.1 (0.6)	42.1 (5.9)	33.7 (0.3)
% Prepeak2		2.2 (0.3)	19.8 (0.4)	47.5 (6.0)	61.7 (0.4)

To assess specificity of the detected mAb3/huFcRn receptor interaction, a triple A mutant of an IgG1 antibody (I266A, H329A, H466A; see material and methods) with abrogated FcRn binding capacity was used as a negative control. Compared to the mass spectra obtained for mAb3 (**Fig 1C**), only a minimal non-specific triple A mutant/huFcRn receptor interaction (**Fig 2C**, marked by asterisks) was observed with the optimized instrument parameters and antibody/FcRn ratios. Interestingly, no higher order aggregates (*m/z* > 9000) were observed for the triple A mutant/FcRn mixed solutions (**Fig 2C**), whereas the formation of FcRn (**Fig 2B**) and antibody (**Fig 2A**) dimers were again detected.

Following the optimization of the system, the native MS approach was applied to study the effect of Met oxidation in the constant IgG1 domains on mAb3/huFcRn receptor interaction.

To induce specific oxidation at the conserved IgG1 heavy chain methionine residues 265 and 459 (HC-Met-265/459; according to Kabat),^45^ we exposed mAb3 to different H_2_O_2_ concentrations (0.003 to 0.02%) for 24 h at 25 °C. No significant effect on mAb3 fragment or aggregate content was observed for all conditions tested as determined by size exclusion chromatography (SEC; results summarized in **Table 3**).The stressed samples were subsequently analyzed by tryptic peptide mapping combined with quantitative ultra-performance liquid chromatography mass-spectrometry (UPLC-MS; **Table 3**). We found increased levels of oxidation for LC-Met-4, HC-Met-100c, and HC-Met-459, whereas oxidation at the buried HC-Met-82^16^ residue was not induced by any of the applied stress conditions. The HC-Met-265 displayed a significant elevation in oxidation (from 4% to 23%) already with 0.003% H_2_O_2_ and was found predominantly oxidized (71%) with 0.02% H_2_O_2_, suggesting that HC-Met-265 represents the most susceptible oxidation site of mAb3. These results are in agreement with previous studies on the sensitivity of Met residues in the constant IgG1 domains.^10,16,19^ In contrast, the degree of asparagine deamidation and aspartate isomerization in the variable and constant IgG1 regions was not significantly affected by the oxidizing stress applied, suggesting that the binding of the mAb3 with huFcRn was not influenced by additional chemical degradation events (**Table 3**).

**Figure 3.**
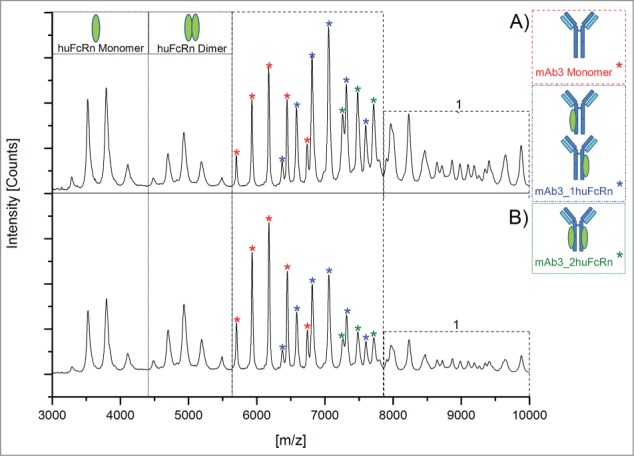
Native MS spectra of mAb3/huFcRn solutions (ratio 1:3) with (**A**) mAb3 reference material and (**B**) oxidized mAb3 (0.02% H_2_O_2_). 1, higher order complexes of mAb3/huFcRn are more abundant in solutions with mAb3 reference material.

Subsequently, functional testing of all oxidized samples was performed by native MS, surface plasmon resonance (SPR) analysis, and huFcRn affinity chromatography. For native MS experiments, oxidized samples were analyzed 5-fold and 3 data sets with comparable resolution and intensity were selected for quantitative data evaluation. **Figure 3** depicts a comparison of the native MS spectra obtained for the mAb3/huFcRn solutions with (A) non-stressed and (B) stressed mAb3 (0.02% H_2_O_2_). The mass spectra and the calculated quantitative data demonstrate that oxidation of mAb3 results in the stepwise reduction of mAb3/huFcRn receptor complex formation and, accordingly, in increased detectable levels of non-complexed mAb3 and huFcRn (**Table 3**). The appearance of higher order aggregates was also reduced in oxidized mAb3 samples (**Fig 3B**). In detail, the reduced intensity of the mAb3/huFcRn receptor complexes with one (from 50% to 42%) or 2 (from 28% to 14%) huFcRn molecules directly correlates to the stepwise increase of oxidation levels at HC-Met-265 (from 4% to 71%; **Table 3**). Consistently, a 22% increase of non-complexed mAb3 was observed. In agreement with the determined oxidation content at the peptide level, huFcRn affinity chromatography verified the successive origination of intact mAb3 variants with one (**Table 3**, Pre-Peak 1) or 2 (Pre-Peak 2) oxidized HC-Met-265 residues (chromatograms are depicted in supplementary data 1). Functional evaluation by SPR analysis also demonstrated a significant loss of mAb3 relative binding capacity (from 100% to 55% relative binding; **Table 3**) to huFcRn (SPR sensorgrams are displayed in supplementary data 2).

**Figure 4.**
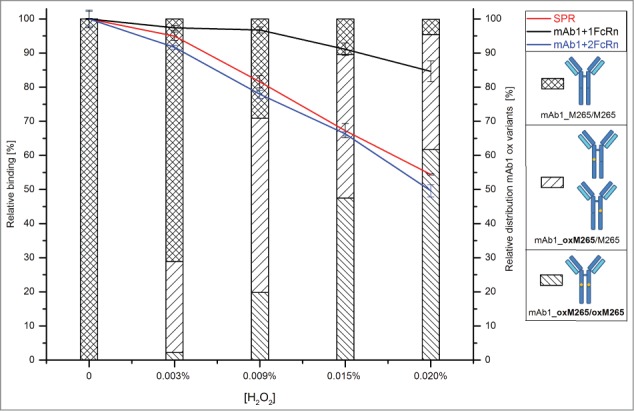
Relative variation in the mAb3/huFcRn receptor complex abundances due to stepwise oxidation of the heavy chain methionine 265 and comparison to the SPR and huFcRn affinity chromatography data. The estimated absolute abundance of mAb3/huFcRn receptor complexes with one or 2 FcRn molecules was set to 100%. A significant impact on relative binding capacity was only detected for mAb3 variants with both heavy chain methionine 265 oxidized.

Initially, it appeared that the observed reduction in the mAb3/huFcRn receptor complex formation in the native MS results (∼22% loss in the summed 1xFcRn Complex and 2xFcRn Complex abundance) did not linearly correlate with the recorded SPR data (45% reduction in relative binding capacity). However, the mass spectrometric data obtained also showed a decrease in the higher order aggregates for the oxidized mAb3 samples (**Fig 3**) indicating that these aggregates do represent higher order mAb3/huFcRn receptor complexes (see discussion section for details). Due to the relatively low resolution of the applied mass spectrometric system, these oligomers could not be unequivocally assigned nor quantified. To compensate for this technical limitation, the relative quantitative variation of the mAb3/huFcRn receptor complexes with one (50%) or 2 (28%) FcRn molecules were normalized to 100%, the relative changes re-calculated and again compared to the SPR and huFcRn affinity chromatography data (**Fig 4**). This approach demonstrated a linear correlation between reduction of mAb3/huFcRn receptor complexes with 2 huFcRn molecules (from 100% to 50%) and the loss of relative binding capacity of mAb3 to huFcRn (from 100% to 55%) as determined by SPR analysis (**Fig 4**). In contrast, no correlation between reduction of mAb3/huFcRn receptor complexes with one huFcRn molecules and SPR data was observed. Moreover, a correlation in the loss of relative binding capacity for the native MS and the SPR data and the appearance of doubly oxidized mAb3 (with both HC-Met-265 oxidized) was detectable, whereas singly oxidized HC-Met-265 (**Fig 4**; at 0.003 % H_2_O_2_) was not found to significantly affect mAb3 binding to huFcRn.

In summary, the functional effect of HC-Met-265 oxidation on mAb3 binding to huFcRn can be evaluated by native MS monitoring of the abundance of mAb3/huFcRn receptor complexes with 2 huFcRn molecules.

## Discussion

For functional characterization of Met oxidation in the constant IgG domains on binding to the huFcRn receptor, a new approach employing a combination of oxidative stress conditions and quantitative LC-MS peptide mapping with native MS was developed. The application of optimized mass spectrometric voltage and pressure conditions significantly stabilized higher *m/z* ions (lower charge states) of the explored mAb3/huFcRn receptor complexes. However, by using the described parameter settings, low levels of FcRn and mAb3 dimer formation were observed. Since the mass range of dimer charge states detected did not significantly interfere with the spectral peaks used for the semi-quantitative analysis of the mAb3 monomer and the mAb3/huFcRn receptor complexes (observed between *m/z* 5500 and 8000), the levels of method-induced artifacts was regarded as tolerable. Moreover, the specificity of the detected mAb3/huFcRn receptor interaction in the native MS approach was verified by the use of a triple A IgG1 mutant with abrogated FcRn binding capacity.

In addition, the appearance of higher order mAb3/huFcRn receptor complexes with mass values beyond 250000 Da (*m/z* > 9000) was also verifiable. Since these higher order aggregates were not observed with the triple A mutant and were significantly reduced with the functionally-impaired oxidized mAb3 samples, we can conclude that the higher order mAb3/huFcRn receptor complex formation are most likely not a method artifact. The generation of higher order oligomeric ribbon forms of mAb3/huFcRn receptor complexes under physiological conditions was already suggested in previous studies on IgG1/ huFcRn receptor interaction.^42-44^ The assignment and relative abundance of these oligomers (consisting of mAb3/2xFcRn) could not be determined with confidence due to the relatively low ionization efficacy and resolution of the mass spectrometric system used. This technical limitation could be partially solved by using a high resolution mass spectrometric system. However, the semi-quantitative evaluation of mAb3/huFcRn receptor complexes versus non-complexed mAb3 is intrinsically impaired by the different ionization efficacies of the different species. Thus, the relative abundance of mAb3/huFcRn receptor complexes with a 1:2 stoichiometry (28%) vs. non-complexed mAb3 (23%) and mAb3/huFcRn receptor complexes with a 1:1 stoichiometry (50%) is definitely underestimated. In addition, our data suggest that mAb3 and huFcRn form a complex with 1:2 stoichiometry (with 2 single FcRn molecules binding to both sides of Fc rather than an FcRn dimer binding to a single site on Fc). Data recently reported by Abdiche et al.^46^ also supports this conclusion. The analysis/resolution of higher order mAb3/huFcRn receptor complexes is also impeded by the inherent glycosylation sites of mAb3 and FcRn (mainly complex-type biantennary oligosaccharides).^47-51^ In order to reduce the complexity caused by the heterogeneous sample population, we independently treated mAb3 and huFcRn receptor with PNGase F prior to native MS analysis (data not shown). However, the tested deglycosylation step did not result in a beneficial higher resolution of higher order mAb3/huFcRn receptor complexes due to incomplete deglycosylation of the huFcRn and was therefore not introduced in our experimental procedure.

The detected charge states for the higher order mAb3/huFcRn receptor complexes did not significantly interfere with the *m/z* range for the mAb3 monomer and mAb3/huFcRn receptor complexes observed (between *m/z* 5500 and 8000). Moreover, the described technical limitation of the MS system was compensated by interpreting the relative quantitative variation of the mAb3/huFcRn receptor complexes (the native MS data of the non-oxidized mAb3 material was set to 100%). This approach demonstrated a linear correlation between the semi-quantitative native MS data of the mAb3/huFcRn receptor complexes with 2 huFcRn molecules and the SPR analysis results of the relative mAb3 binding capacity to huFcRn.

Subsequently, different H_2_O_2_ concentrations were used to study the effect of Met oxidation in the constant IgG1 domains on the mAb3/huFcRn receptor interaction. Quantitative UPLC-MS analysis demonstrated that HC-Met-265 represents the most susceptible oxidation site of mAb3, whereas LC-Met-4, HC-Met-82, HC-Met-100c, and HC-Met-459 were found to be less prone to oxidation. These results are in agreement with previous studies on the sensitivity of Met residues in the variable and constant IgG1 domains.^10,16,19^ It should be noted that ionization efficiencies of peptides can differ significantly depending on their amino acid content. Increasing hydrophobicity usually leads to an enhanced formation of ionic species. On the other hand variation of the pI value of peptides also alters the ionization efficiency. Consequently, different ionization efficiencies have to be taken into account when discussing and interpreting the data obtained from the presented experiments. However, the detected levels of oxidized tryptic peptide species for HC-Met-265 do correspond/agree with the data obtained here from huFcRn affinity chromatography and the relative binding capacity of oxidized mAb3 to huFcRn (as determined by SPR analysis) suggesting that the oxidation content reported is not significantly overestimated for the peptide species (**Table 3**). Since no alterations in asparagine deamidation, aspartate isomerization, and structural integrity were observed, the stress condition approach with H_2_O_2_ was determined to be suitable for the generation of specific mAb3 oxidation variants.

Oxidation of HC-Met-265 in recombinant IgG1 antibodies has been demonstrated to affect the in vitro interaction with huFcRn receptor.^9,24^ In addition, a clear effect of HC-Met-265 oxidation on the pharmacokinetics in transgenic mice models has been reported in 2 independent in vivo studies.^13,25^ By using isolated huFcRn affinity chromatography fractions of oxidized IgG1, Stracke et al. also elucidated that only oxidation at HC-Met-265 (but not HC-Met-459 oxidation) significantly alters the FcRn binding properties of IgG1 antibodies. In our study, oxidation of HC-Met-265 resulted in the stepwise reduction of mAb3/huFcRn receptor complex formation. To elaborate, a linear correlation between the decrease in mAb3/huFcRn receptor complex formation with a 1:2 stoichiometry and the loss of relative binding capacity of mAb3 to huFcRn (as determined by SPR analysis) was observed, demonstrating that native MS is suitable for studying the functional effect of antibody oxidation in vitro.

Moreover, and maybe even more interestingly, a quantitative effect of HC-Met-265 oxidation on relative binding capacity was only detected for doubly oxidized mAb3 (with both HC-Met-265 oxidized), whereas mAb3 with a singly oxidized HC-Met-265 was not found to significantly affect mAb3 binding to huFcRn. These results are in agreement with the previous study by Stracke et al. where only IgG1 fractions with both heavy chains oxidized at Met-265 exhibited significantly faster clearance in an FcRn-transgenic mice model.^13^ The significantly altered binding affinity of the probed mAb3 to huFcRn is presumably due to the fact that only fully oxidized Met-265 significantly changes the conformation of the monoclonal antibody Fc fragment.^12,52^ Thus, mono-oxidized IgG1 HC-Met-265 most likely does not represent a critical quality attribute.

In summary, the application of specific stress conditions combined with native MS, quantitative UPLC-MS peptide mapping, and functional evaluation by SPR was adequate to identify and functionally assess Met oxidation in the constant IgG1 domains. Moreover, the whole multimeric IgG1–FcRn complex in solution was verified for the first time by native MS. The reported native MS methodology and results might also be of importance for studying antibody-antigen interactions and for other major classes of biopharmaceuticals such as Fc-fusion proteins, protein scaffolds, and bispecific antibodies.^29,53,54^

## Materials and Methods

### Sample preparation and oxidative stress conditions

The recombinant IgG1 antibody mAb3 was expressed in a Chinese hamster ovary cell system. The antibody was manufactured at Roche Diagnostics, Penzberg, Germany using standard cell culture and purification technology. Cloning, expression, and purification of huFcRn receptor and triple A (I253A, H310A, H435A) IgG1 mutant was performed as previously described.^24^ mAb3 was formulated at a concentration of 15 mg/mL in a His-HCl buffer system (20 mM His-HCl, 100 mM NaCl) at pH 6.0. Oxidation of mAb3 methionine residues was performed using different H_2_O_2_ concentrations (ranging from 0.003% to 0.02%; v/v) in the same buffer system for 24 h at 25°C. Removal of the oxidizing reagent was performed using NAP™-5 gel filtration columns (GE Healthcare, #17-0853-02). Samples were then fractionated and frozen at −80°C until further analysis.

### Tryptic peptide mapping

For the detection and quantification of Asn deamidation, Asp isomerization and Met oxidation at peptide level, mAb3 was denatured in 0.2 M His-HCl, 8 M Gua-HCl, pH 6.0 by diluting 350 µg of mAb3 in a total volume of 300 µL. For reduction, 10 µl of 0.1 g/mL dithiothreitol (DTT) was added followed by incubation at 50°C for 1 hour. Next, the buffer solution was exchanged to a digestion buffer (0.02 M His-HCl, pH 6.0) using NAP™-5 gel filtration columns. Subsequently, the NAP™-5 eluate (500 µL) was mixed with 10 µL of a 0.25 mg/mL trypsin solution (Trypsin Proteomics grade, Roche, #03708985001) in 10 mM HCl and incubated at 37°C for 18 h.^26^

### Analysis of proteolytic peptides by liquid-chromatography mass-spectrometry

The tryptic peptide mixture was separated by RP-UPLC (ACQUITY, Waters, Manchester, UK) on a C18 column (BEH C18 1.7 µm, 2.1 × 150 mm; Waters) and analyzed online with a Synapt G2 electrospray mass spectrometer (Waters). The mobile phases consisted of 0.1% formic acid in water (solvent A) and 0.1% formic acid in acetonitrile (solvent B). The chromatography was carried out using a gradient from 1 to 35% solvent B in 45 min and finally from 35 to 80% solvent B in 3 min using a flow rate of 300 µL/min. UV absorption was measured at a wavelength of 220 nm. 3.5 µg digested protein was applied. The UPLC-system and mass spectrometer were connected by PEEK-capillary tubes. Data acquisition was controlled by the MassLynx software (Waters). Parameters for MS detection were adjusted according to general experience available from peptide analysis of recombinant antibodies.

### Data analysis for the quantification of chemical modification levels

Peptides of interest were identified by searching manually for their *m/z* values within the mass spectrum. For the quantification, specific ion current chromatograms of peptides of interest were generated on the basis of their monoisotopic mass and detected charge states using GRAMS AI software (Thermo Scientific, V8.0). Relative amounts of Asn deamidation, Asp isomerization, and Met oxidation were calculated by manual integration of modified and unmodified peptide peaks.

### Preparation of mAb3/huFcRn receptor complex samples

Prior to native MS analysis, 300 µl of huFcRn receptor solution (0.8 mg/mL) was washed thrice with 500 µl PBS (1 mM KH_2_PO_4_, 10 mM Na_2_HPO_4_, 140 mM NaCl, 3 mM KCl, pH7.0) using prewashed 5K Vivaspin® (Satorius Stedim Biotech GmbH, #VS0112) centrifugal devices (10 min at 12.000 g) to remove storage buffer. The supernatant was then diluted with water to a final volume of 300 µl. Buffer exchange to 50 mM ammonium acetate buffer (pH 6.0) was performed using NAP™-5 gel filtration columns. Simultaneously, 765 µg of mAb3 reference material or freshly oxidized material was also buffer exchanged to 500 µl 50 mM ammonium acetate (pH 6.0) using again NAP™-5 gel filtration columns. The protein concentration for all samples generated was determined by conventional UV photometry (Uvikon XL, GOEBEL Instrumentelle Analytik GmbH, Au, Germany). Subsequently, all FcRn and mAb3 samples were diluted with 50 mM ammonium acetate buffer (pH 6.0) to a final concentration of 8 µM, respectively. A mAb3/huFcRn solution with a ratio of 1:3 was freshly prepared and incubated at RT for 30 min prior to native mass spectrometric analysis.

### Native mass spectrometric analysis of mAb3/huFcRn receptor complexes

Analysis of mAb3/huFcRn complexes was performed on a Q-TOF Ultima mass spectrometer system (Waters) upgraded by MS Vision (Almere, The Netherlands) as a High Mass Q-TOF, which enables measurements of protein/protein complexes at higher mass ranges. Samples were introduced into the MS system using Advion's nanomate direct infusion system (Ithaca, NY, USA). All samples were analyzed using the optimized MS parameters (summarized in **Table 1**), which allowed adequate detection of mAb3/huFcRn complexes. Cone voltage was set at 45 V, RF Lens1 at 150 V and collision energy to 20 V. The vacuum in the collision cell was adjusted to 1.10 e^−2^ mbar. Additionally, the source vacuum was set to 2.5–2.7 bar resulting in vacuum values for the mass analyzer of 1.42 e^−4^ and 7.42 e^−7^ for the TOF Penning. Individual samples were 5-fold analyzed for 20 min. Three of the recorded data sets were further quantitatively analyzed after applying the following spectra selection criteria: resolution for the non-complexed mAb3 charge states had to be comparable between selected data sets to estimate correct relative quantification values.

### Data analysis for the relative quantification of FcRn/antibody complexes

For calculating the relative ratios of non-complexed mAb3 versus mAb3/huFcRn complexes with 1 or 2 huFcRn receptors the following charge states were selected. Non-complexed mAb3: charge states +23 to +25. mAb3/huFcRn complex with one huFcRn receptor: charge states +27 to +29. mAb3/huFcRn complex with 2 huFcRn receptors: charge states +32 to +34. Non-complexed huFcRn receptor was not selected for the quantitative analysis. Area calculation for the selected charge states was conducted by manual integration using GRAMS AI software. Relative amounts of non-complexed mAb3 vs. mAb3/huFcRn complexes with one or 2 huFcRn receptors were calculated by averaging and direct comparison of the peak areas recorded for the selected charge states. All selected charge states for mass determination and subsequent semi-quantitative functional analysis are summarized in **Table 2**.

### Size exclusion chromatography

SEC was carried out using a TSK-Gel G3000SWXL column (7.8 × 300 mm, 5 µm particle size; Tosoh Bioscience, Amsterdam, Netherlands). An isocratic elution using 100 mM KH_2_PO_4_, pH 6.8 at 0.5 mL/min as solvent was used for chromatographic separation on an Ultimate3000 HPLC-system equipped with UV detection at 280 nm. 200 µg of mAb was injected for the chromatographic analysis and data acquisition was controlled by Chromeleon software (Dionex Softron GmbH, Germering, Germany). Relative quantification was performed by manual integration and comparison of peak areas.

### Surface plasmon resonance analysis – FcRn binding

The interaction between the stressed or non-stressed mAb2 and the specific target protein was measured by SPR using a Biacore T200 instrument (GE Healthcare). To evaluate mAb interaction to its specific target, a relative active concentration assay was performed. The specific target protein was immobilized onto a Biacore CM5-biosensor chip (GE Healthcare) via amine coupling to reach a coupling density of about 3500 RU. The assay was carried out at room temperature with PBS-P + pH 6.0 buffer (GE Healthcare) as running and dilution buffer. 50 nM of native or stressed mAb2 samples were injected at a flow rate of 30 µL/min. Association time was 200 s, followed by a 300 s dissociation phase. Regeneration of the chip surface was reached by a 30 s injection of PBS-P + pH 8.0. Evaluation of SPR-data was performed by comparison of the response of samples and reference material 5 seconds before the end of the association phase. Activity of reference material was set to 100%.

### Analytical FcRn chromatography

Separation and sample homogeneity after the preparative FcRn chromatography runs were confirmed for all 5 samples by analytical scale FcRn affinity chromatography. For this, FcRn-sepharose matrix was filled in a MonoQ column housing (inner diameter 5 mm × length 50 mm, GE Healthcare) and incorporated in a HPLC system with UV detector. The column was equilibrated with 20 mM 2-(N-morpholine)-ethanesulfonic acid (MES) buffer containing 150 mM NaCl, pH 5.5 (eluent A) at 0.3 ml/min. Fifty μg of each sample were diluted with eluent A and applied to the FcRn column at room temperature. The column was washed with 5 column volumes of eluent A followed by elution in a linear gradient from 0–100% 20 mM Tris/HCl, 150 mM NaCl, pH 8.5 (eluent B) in 36 column volumes. The elution profile was obtained by continuous measurement of the absorbance at 280 nm.
