# Supplementary material for: Functional assessment of antibody oxidation by native mass spectrometry
Source: MAbs. 2015 May 22;7(5):891–900. doi: 10.1080/19420862.2015.1052199 (PMC4622615; doi:10.1080/19420862.2015.1052199)

**Supplementary Files: Figure Legends**

Fig. 1:

Overlay of the analytical FcRn chromatograms of non-stressed mAb3 (stored at -80°C) versus stressed material. (*), heavy chain methionine 265 oxidation.

Fig. 2:

Analysis of mAb3 huFcRn binding by surface plasmon resonance. Biacore sensorgrams showing the huFcRn binding of non-stressed mAb3 material (stored at -80°C) and following oxidative stress conditions (zoom in view from 10-350 seconds acquisition time).

**Supplementary Figure 1:**


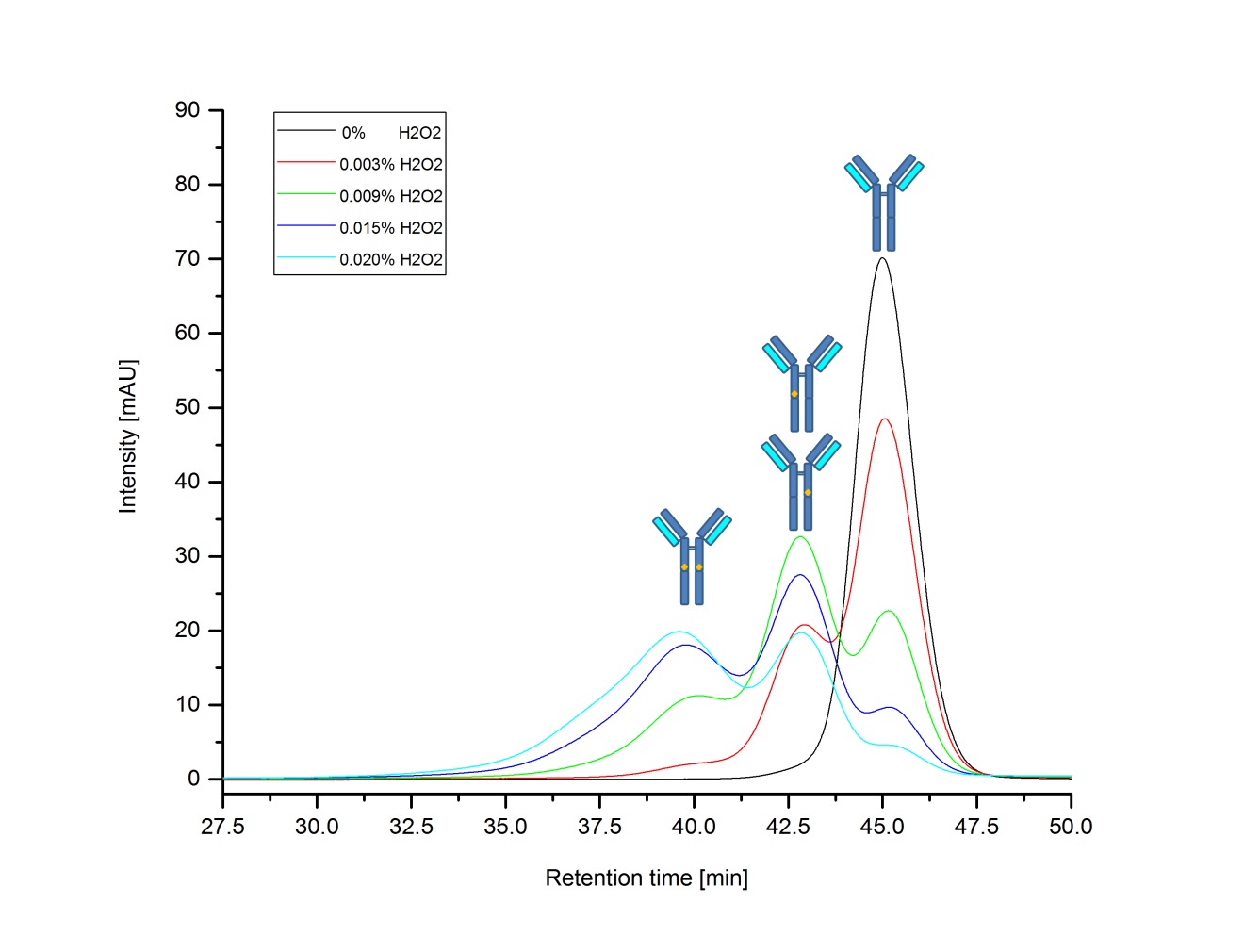


**Supplementary Figure 2:**


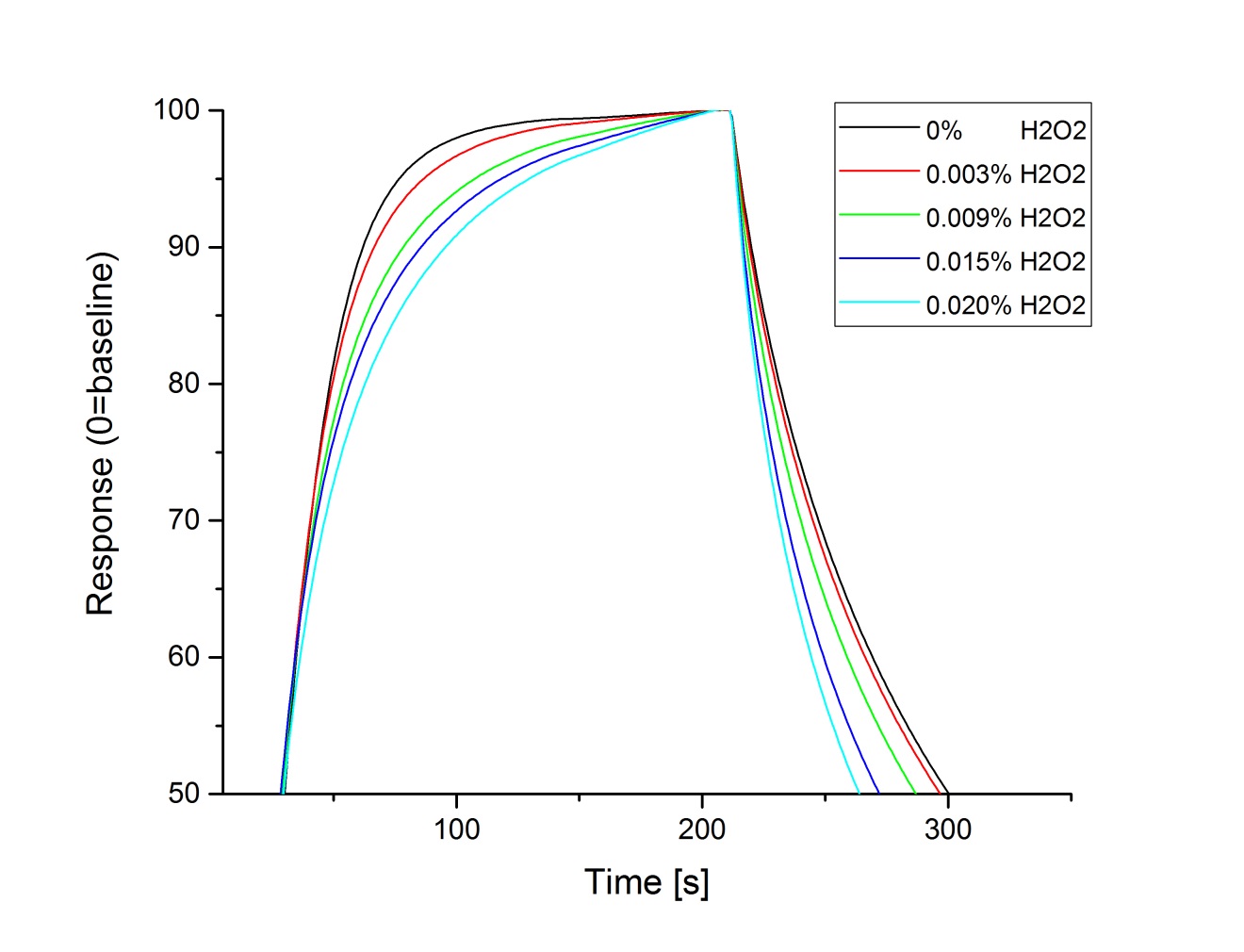

Supplement: Supplemental_Material.docx [file kmab-07-05-1052199-s001.docx]
